# CLGBO: An Algorithm for Constructing Highly Robust Coding Sets for DNA Storage

**DOI:** 10.3389/fgene.2021.644945

**Published:** 2021-05-04

**Authors:** Yanfen Zheng, Jieqiong Wu, Bin Wang

**Affiliations:** The Key Laboratory of Advanced Design and Intelligent Computing, Ministry of Education, School of Software Engineering, Dalian University, Dalian, China

**Keywords:** DNA storage, primer and address sequences, CLGBO, non-adjacent subsequence constraint, DNA coding sets

## Abstract

In the era of big data, new storage media are urgently needed because the storage capacity for global data cannot meet the exponential growth of information. Deoxyribonucleic acid (DNA) storage, where primer and address sequences play a crucial role, is one of the most promising storage media because of its high density, large capacity and durability. In this study, we describe an enhanced gradient-based optimizer that includes the Cauchy and Levy mutation strategy (CLGBO) to construct DNA coding sets, which are used as primer and address libraries. Our experimental results show that the lower bounds of DNA storage coding sets obtained using the CLGBO algorithm are increased by 4.3–13.5% compared with previous work. The non-adjacent subsequence constraint was introduced to reduce the error rate in the storage process. This helps to resolve the problem that arises when consecutive repetitive subsequences in the sequence cause errors in DNA storage. We made use of the CLGBO algorithm and the non-adjacent subsequence constraint to construct larger and more highly robust coding sets.

## Introduction

**FIGURE 1:**
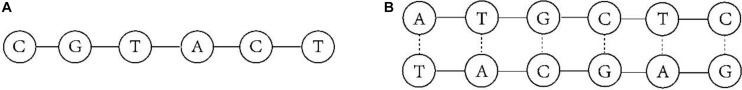
DNA model. **(A)** single-stranded DNA. **(B)** double-stranded DNA.

The amount of global data has exponentially increased with the advent of the Internet age, and is expected to grow from 45 ZB in 2019 to 175 ZB in 2025 (Reinsel et al., 2018). The problems of existing storage media, which include difficulty in achieving large capacity storage, the existence of high maintenance costs, limited service life and easy data loss, mean that the storage industry faces unprecedented challenges and opportunities (Zhirnov et al., 2016). It is therefore urgent to find a new storage medium to meet the demand of data storage. In 1953, Watson and Crick (1953) published a paper on the molecular structure of nucleic acid. Their research opened the door to the study of biogenetics, and also promoted people to begin exploring the form of life from a new perspective at the molecular biological level. Later, deoxyribonucleic acid (DNA) molecular replication, DNA molecular recombination, genetic code, genetic information transmission and other genetic molecular mechanisms make people have a more comprehensive and profound understanding of DNA gene theory. Information about organisms has been stored in DNA molecules composed of four bases called adenine (A), thymine (T), guanine (G), and cytosine (C) for three billion years since life first came into existence on the earth. Pair pairs between A and T, C, and G can form stable double-stranded structures, and both single-stranded DNA and double-stranded DNA can be used to store information in the form of binary code. Figure 1 shows the structural models of single-stranded and double-stranded DNA. DNA storage has the advantages of high storage density, low maintenance costs and long storage life compared with the traditional storage media, and it is a widely studied area for researchers (Ping et al., 2019).

In 2012, Church et al. (2012) successfully stored a 650 KB sized book in oligonucleotides (shorter DNA sequences) and retrieved them by sequencing. Shortly thereafter, Goldman et al. (2013) stored 739 KB of information in DNA and recovered the original file with 100% accuracy. In 2015, Grass et al. (2015) demonstrated that digital information could be stored in DNA and that the original information could be recovered error-free over long periods of time using error-correcting codes. Later, in the same year, Yazdi et al. (2015) proposed that DNA storage could provide ultra-high data storage capacity. They described a DNA storage architecture that allowed random access and rewriting of information blocks. In 2017, Erlich and Zielinski (2017) stored a complete computer-operating system, movies and other files in a DNA sequence with a total size of 2.14 × 10^6^ bytes. This level of storage was several orders of magnitude higher than previously reported work that used a storage strategy called DNA Fountain. In 2018, Organick et al. (2018) stored 35 different files (over 200 MB of data) and demonstrated that each file could be recovered accurately using a random-access method. A year later, Lopez et al. (2019) demonstrated the successful decoding of 1.67 MB of information stored in DNA sequences using portable nanopore sequencing. In 2020, Meiser et al. (2020) proposed a protocol that focused on providing an ideal starting point for small experiments and reducing the corresponding error rate by changing the parameters of the encoder/decoder to achieve a higher amount of data storage and random access to the data. Chen Y. J. et al. (2020) studied the heterogeneity of oligonucleotide replication and showed that the two main sources of bias were the synthesis and amplification processes. They built statistical models for each molecule and the entire process based on these findings. Lin et al. (2020) proposed a simple architecture consisting of a T7 promoter and a single-strand protruding domain (SS-dsDNA) that can be used for dynamic DNA information storage. In another study (Chen H. et al., 2020), Chen et al. proposed a DNA hard drive as a rewritable molecular storage system. Data could only be read after the correct key was provided, which ensured the security of the data storage. In 2021, Cao et al. (2021) proposed a thermodynamic minimum free energy constraint and applied to the construction of DNA storage coding sets. The introduction of this constraint improves the quality of DNA coding and reduces the error rate in the storage process.

The process of DNA storage involves the following steps: DNA coding (mapping binary data to nucleotide sequences), DNA synthesis (synthesizing specific sequences of DNA to complete the writing of the code), DNA processing and storage, polymerase chain reaction (PCR) amplification to enable random access to the data, followed by sequencing (reading) with a sequencing instrument, and DNA decoding (mapping nucleotide sequences to binary data).

Three important processes in the DNA storage are described here in detail. The results of DNA coding directly affect the performance of DNA storage. The entire DNA coding consists mainly of the process of data compression, introduction of error correction and conversion to DNA sequence:

(1) Compression: Compression makes greater use of DNA storage space and removes redundancy before storing information in DNA. Common compression methods include Hoffman coding and Fountain coding but there are many examples. In 2013, Goldman et al. (2013) used Hoffman coding in DNA storage for the first time, which increased the coding efficiency to 1.58 bit/nt. This coding method can reduce but does not avoid the appearance of homopolymers; it does control the GC content well. In 2017, Erlich and Zielinski (2017) used DNA Fountain in DNA storage for the first time and used a quadratic conversion model with 00, 01, 10, and 11 mapped to A, C, G, and T, respectively. This encoding method filters sequences containing homopolymers and GC content anomalies and improves the encoding efficiency to 1.98 bit/nt.
(2) Introduction of error correction: In each process of DNA storage, errors may occur that result in the loss of the original digital information. The introduction of an error-correction mechanism is necessary to obtain accurate information. The introduction of an error-correction mechanism at the coding stage is the most effective way to ensure accuracy and cost saving. Error correction improves accuracy by removing redundancy. It is, however, critical to strike a balance between redundancy and accuracy. At present, Reed-Solomon codes (RS codes) are the main error-correction method. In 2015, Grass et al. (2015) applied RS coding to DNA storage for error-free storage. RS coding has the advantage of recovering more information about the original data with less redundancy.
(3) Conversion model: The conversion of digital information to DNA information is required for the conversion model to work. The coding model can be divided into three forms depending on the conversion method: binary, ternary, and quaternary. The binary model was used by Church et al. (2012) in 2012. The conversion of the model was achieved by encoding the binary digits into specific DNA sequences where A or G was coded as 0 and T or C was coded as 1. The binary model effectively avoids the effects of unbalanced GC content or homopolymers. In 2013, Goldman et al. (2013) used the ternary model to convert information into DNA sequence. The entire base sequence had three states: 0, 1, and 2. The ternary model mainly determines the last base by the first base. However, it does not establish a specific mapping relationship between bases and data like the binary model. The ternary model can store more information than the binary model. However, the ternary model does not take full advantage of the storage power of DNA. In 2017, Erlich and Zielinski (2017) used the quaternary coding model to map A, T, G, and C to 00, 01, 10, and 11, respectively. The quaternary coding model has the strongest storage capacity compared with other models, but it is prone to excessive GC content and high homopolymers, which impacts DNA storage.

Deoxyribonucleic acid synthesis chemically joins one nucleotide to another and forms a single-stranded DNA sequence (Kosuri and Church, 2014). In the synthesis process, the coupling efficiency is 99% at each step but the small error still results in an exponential decrease in product yield with increasing length. Therefore the length of the synthesized DNA sequence should be kept to about 200 nucleotides (Bornholt et al., 2016). DNA sequencing technology is used to determine the DNA sequence. The current DNA sequencing technology is divided into three main generations. The first generation of sequencing technologies mainly include the double deoxygenated strand end-termination sequencing method proposed by Sanger et al. (1977) and the chemical degradation method invented by Maxam and Gilbert (1977). The first generation DNA sequencing technologies can sequence up to 1,000 bp in length, but have the disadvantages of slow speed (the automatic Sanger sequencer can only read 1,000 bases in 24 h) and high cost (about $1 to sequence 600–700 bp). The second generation of sequencing technologies arose due to advances in science and technology and the efforts of researchers to specifically improve sequencing technologies. It is also known as next generation sequencing (NGS) or high throughput sequencing, which allows rapid sequencing of millions of molecules simultaneously at one time. The second generation of sequencing technologies also has its limitations. Most NGS requires primers for *in vitro* template amplification and sequencing of the resulting template library. Replication errors and loss of information can occur during this process (e.g., the errors mentioned earlier are most likely to occur in sequences with high and low GC content and the presence of homopolymers; Church et al., 2012). The second generation of sequencing technologies solved the problem of high throughput. Today researchers are more inclined to study the characteristics of single molecules of DNA and the third generation of DNA sequencing technology was created for this purpose. The third generation sequencing refers to single-molecule sequencing technology. It is capable of analyzing long sequences and produces only random errors although it has a relatively high error rate (about 10%; Yazdi et al., 2017). It is an inevitable that DNA storage technology will be widely used in the next few years due to the maturity and success of DNA synthesis and sequencing technologies (Carmean et al., 2018). However, non-specific hybridization, mutation, insertion, deletion and other errors are common during DNA storage and that can lead to data-reading errors and deletions.

Therefore, it is vital to study the sources of errors that impact DNA storage and coding. Earlier studies (Myers, 2007 Tandem Repeats and Morphological Variation | Learn Science at Scitable; Kovacevic and Tan, 2018; Schwarz et al., 2020) revealed that the error rate in the storage process increases if there are consecutive repetitive subsequences in the sequence. Hence, we propose a novel constraint (non-adjacent subsequence constraint) to avoid the occurrence of this sequence. The design of coding sets under multiple constraints is difficult and belongs to the NP problem. However, the heuristic algorithms that have emerged in recent years are well suited to this problem by virtue of their low complexity and high accuracy. Hence, an improved optimization algorithm is proposed, which uses two mutation strategies to enhance the gradient-based optimizer (GBO). Specifically, this algorithm takes advantage of the Cauchy mutation operator for random perturbation to increase the diversity of the population and improve the ability of the algorithm to explore the optimal value globally. At the same time, the Levy mutation operator is used to enhance the local search ability of the algorithm, and this helps to avoid falling into local optima. In this study, the combination of Cauchy and Levy mutation strategy (CLGBO) and specific constraints (Hamming distance, GC content, No-runlength constraint, and non-adjacent subsequence constraint) not only ensures the quality of the coding sets but also improves its lower bounds.

The article is structured as follows: section “Coding constraints in DNA storage” describes in detail the four constraints of the coding sets in DNA storage. Section “Algorithm description” describes the CLGBO algorithm and the test results and analysis of the improved algorithm. Section “Designing of lower bounds of coding sets” describes the design of coding sets and comparison of lower bounds of DNA coding sets. Section “Conclusion” summarizes this study and presents an overall outlook.

## Coding Constraints in DNA Storage

The most important and difficult aspect of DNA storage is the synthesis and sequencing of DNA strands. The two processes are most prone to substitution, deletion and insertion errors. According to statistics, the error probability of each nucleotide in the sequencing process is 1% (Press et al., 2020), and some special cases (For example, there are homopolymers, consecutive repetitive subsequences, and the content of G and C bases is too high or too low in the DNA sequence.) will produce higher error rate. During storage, DNA molecules are prone to non-specific hybridization reactions. If non-specific hybridization occurs between DNA molecules, it may prevent the DNA molecules carrying information from being sequenced normally, and will also cause data reading failure. By restricting the DNA sequence to comply with the following constraints, the incidence of non-specific hybridization and the rate of read and write errors can be reduced:

### Non-adjacent Subsequence

Deoxyribonucleic acid sequences containing consecutive repetitive subsequences are more likely to be misaligned during sequencing and this results in data-reading errors (Myers, 2007 Tandem Repeats and Morphological Variation | Learn Science at Scitable). Sequences containing consecutive repetitive subsequences easily produce polymerase slippage at the synthesis phase (Schwarz et al., 2020). Two DNA sequences can easily become dislocated in the repetitive region. For example, an ATG subsequence on one sequence could base-pair with the first TAC in the other sequence, or the second, or the third. In this study, we mainly focus on the case where the length of subsequence is 2 and 3. For example, there is a subsequence AG in the GTAGAGAGCTA sequence, and there is a subsequence TGA in the AGTGATGACG sequence. Sequences containing these two types are not added to the DNA coding sets. For the coding set A, any sequence S (S = *s_1_s_2_*…*s*_*n*_) exists as follows:

(1) $$\begin{array}{c} \begin{array}{cc} w\,h\,e\,n & K=2 \end{array}\\ \begin{array}{ccc} & & \begin{array}{cccc} & & & \begin{array}{cc} {\text{s}}_{\text{i}}\,s_{i+1}\neq s_{i+2}\,s_{i+3}, & 0 \end{array}<i\leq n-\left(2\,k-1\right) \end{array} \end{array}\\ \begin{array}{cc} w\,h\,e\,n & K=3 \end{array}\\ \begin{array}{ccc} & & \begin{array}{cccc} & & & \begin{array}{cc} {\text{s}}_{\text{i}}\,s_{i+1}\,s_{i+2}\neq s_{i+3}\,s_{i+4}\,s_{i+5}, & 0 \end{array}<i\leq n-\left(2\,k-1\right) \end{array} \end{array} \end{array}$$

### Hamming Distance

For any two sequences *v* (*v* = *v_1_v_2_*…*v*_*n*_) and *u* (*u* = *u_1_u_2_*…*u*_*n*_) of length *n* in the DNA coding sets, the Hamming distance is expressed as the number of different elements at the same position between the two sequences *v* and *u* (Aboluion et al., 2012). H (*v*, *u*) is required to be H (*v*, *u*) ≥ *d*, where *d* is the defined threshold, and H (*v*, *u*) calculates the Hamming distance by the following formula:

(2) $$\begin{array}{cc} H\,\left(v,u\right)=\sum_{i=1}^{n}h\,\left(v_{i},u_{i}\right), & h\,\left(v_{i},u_{i}\right)=\left\{ \begin{array}{cc} 0, & v_{i}=u_{i}\\ 1 & v_{i}\neq u_{i} \end{array}\right. \end{array}$$

The Hamming distance can be used to measure the similarity between different sequences. The larger the value of *d*, the greater the differences between the sequences and the less similar they are. The smaller the value of *d*, the smaller the differences between the sequences, the greater the similarity and the more likely it is for non-specific hybridization to occur between sequences. This will result in storage errors. In addition, the Hamming distance has an error-correction function with relational data elasticity, which can also effectively decrease the error rate in the process.

### GC Content

GC content is the percentage of bases *G* and *C* in a DNA sequence (Wang et al., 2020). An appropriate GC content is crucial to maintain the chemical stability of DNA sequences because the base pair G-C contains three hydrogen bonds, while the base pair A-T contains two hydrogen bonds. Previous work has shown that 50% GC content is optimal (Chee and Ling, 2008; Aboluion et al., 2012; Tulpan et al., 2014) and the formula is as follows:

(3) $$\text{GC}\,\left(s\right)=\frac{\left|G\right|+\left|C\right|}{\left|s\right|}\times 100\%$$

where GC(*s*) denotes the GC content of sequences, | *G*| and | *C*| denote the number of bases *G* and *C*, respectively, in sequence s, and | *s*| denotes the number of bases in the entire sequence.

### No-Runlength

The presence of homopolymers in sequences is one of the major sources of errors during DNA storage. Overly long homopolymers can lead to insertion, substitution and deletion errors (Church et al., 2012). For example, in TAAAGC, the presence of A base repeats can easily be misinterpreted as TAAGC or TAAAAGC during sequencing. Therefore, it is required that each DNA sequence should not contain consecutive repetitive bases (Erlich and Zielinski, 2017). The presence of consecutively repetitive bases during sequencing will read them as a single signal and may result in data loss. It is therefore strictly forbidden to have the same bases adjacent to each sequence and this is mathematically modeled as follows:

(4) $$\begin{array}{cc} S_{i}\neq S_{i+1} & ,i\in \left[1,n-1\right] \end{array}$$

## Algorithm Description

### Gradient-Based Optimizer

Generally speaking, optimization methods can be divided into two categories: one is gradient-based optimization methods, such as gradient descent method (Keshavan and Sewoong, 2009), newton method (Agarwal et al., 2006), and quasi-newton method (Broyden, 1970); the other is non-gradient-based optimization method, namely metaheuristic algorithm. Algorithms of this type can be divided into two categories: one is single objective algorithm such as animal migration optimization algorithm (Li et al., 2014), simulated annealing algorithm (Rutenbar, 1989), cuckoo search algorithm (Li and Yin, 2015), the gray wolf optimization algorithm (Mirjalili et al., 2014), differential evolution algorithm (Li and Yin, 2011a), henry gas optimization algorithm (Hashim et al., 2019), multi-search differential evolution algorithm (Li et al., 2017b), hybrid differential evolution with biogeography-based optimization (Li and Yin, 2011b), the other is multi-objective algorithm such as: the NSGA-II (Huang et al., 2010), multi-objective biogeography based optimization algorithm (Li and Yin, 2013), new multi-objective optimization algorithm combined with opposition-based learning (Ewees et al., 2021), and multi-objective ranking binary artificial bee colony algorithm (Li et al., 2017a).

Ahmadianfar et al. (2020), inspired by the gradient-based Newtonian approach, developed the GBO, a powerful and efficient algorithm that combines gradient and metaheuristic algorithm. Gradient-based methods are widely used to solve optimization problems. The optimal solution using a gradient-based optimization algorithm is found by determining an extreme point at which the gradient is equal to zero. In the gradient-based optimization method, a search direction is selected and the search process moves along this direction toward the optimal solution (Shahidi et al., 2005). In the metaheuristic algorithm, the initial solution (i.e., the initial population) is randomly generated and the search direction is determined from the results of previous searches. The search direction will not stop updating until the convergence condition is satisfied. This kind of method is very effective in finding the global optimal. Therefore, it is worthwhile to develop a population-based optimization algorithm that uses the gradient method to skip infeasible points and move toward feasible regions. GBO is mainly composed of a gradient search rule (GSR) and a local escape operator (LEO). The GSR uses a gradient-based approach to enhance the exploration capability of the algorithm and speed up convergence to obtain a better position in the entire search space. The LEO are mainly used to improve the efficiency of GBO for solving complex problems and to escape from local optima. All detailed mathematical models of GBO can be found in the literature (Ahmadianfar et al., 2020).

### Improved Algorithm

The GBO algorithm has low computational complexity and a simple structure. However, this algorithm also has some disadvantages. The main function of the LEO phase of the algorithm is to avoid the occurrence of local optimal stagnation, but only when the random number is less than 0.5, will it enter the LEO phase (Ahmadianfar et al., 2020). In addition to being easy to fall into the local optimum, the GBO algorithm has the shortcomings of premature convergence, imbalance between exploitation and exploration, and slow convergence speed. A method called mutation strategy is introduced in this work to solve these shortcomings. The basic GBO algorithm is embedded with two innovations, the Cauchy mutation strategy and the Levy mutation strategy, to improve the overall optimization performance of the algorithm. Mutation strategy is a commonly used method for evolutionary algorithms to produce new individuals, which can effectively enrich the population. However, it is difficult for a single mutation operator to effectively balance the exploration and exploitation capabilities of the algorithm. Therefore, an algorithm combining two mutation operators is proposed to alleviate the lack of population diversity, the imbalance between exploitation and exploration, and the premature and slow convergence of the GBO algorithm. The fitness of the mutated individual is compared with the fitness of the parent. The parent is replaced by the mutated individual to improve the overall quality if the fitness of the mutated individual is better than the parent. The experimental results show that the CLGBO algorithm is significantly improved in terms of convergence speed, stability and seeking accuracy.

#### Cauchy Mutation Strategy

**FIGURE 2:**
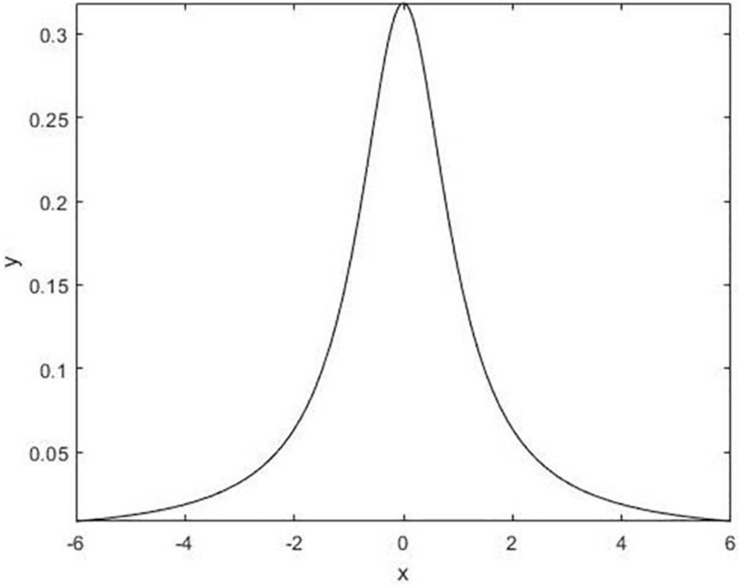
Standard Cauchy distribution density function.

The Cauchy mutation operator is an effective strategy to improve the algorithm (Wang et al., 2007; Hu et al., 2009; Ali and Pant, 2011; Sapre and Mini, 2019). The theoretical basis of the Cauchy mutation operator is derived from the standard Cauchy distribution density function, which is defined by eq. (5). The Cauchy distribution density function has a smaller peak at the origin but a longer distribution at both ends (Figure 2). This allows individuals to have a higher probability of jumping to a better position, which means that the Cauchy mutation operator has strong global control. The Cauchy distribution function has a relatively small peak value and individuals spend less time searching adjacent intervals in the iterative process. More energy is put into searching for the global optimal value around the best individual, which means that the improved algorithm has a good ability to adjust and to optimize its searching capabilities. The use of the Cauchy mutation operator for random perturbation has several benefits. It helps to increase the diversity of the population and makes the exploration range of the previous iteration broader and more inclined to be a promising area. And the important point is that it can effectively reduce the search blind spots and improve the exploration ability of the algorithm. In addition, the characteristics of the Cauchy distribution enable it to generate random numbers that are far away from the origin. This means that individuals after the Cauchy mutation have the ability to quickly escape from the local optimal value. Eqs (5, 6) are given by

(5) $$f_{\text{cauchy}}\,\left(r\right)=\frac{1}{\pi \,\left(1+\gamma^{2}\right)},$$

(6) $$y=\frac{1}{2}+\frac{1}{\pi }\,\arctan\left(\gamma \right).$$

Equation (6) is the mathematical model of the standard Cauchy distribution function and *y* is a random number uniformly distributed on the interval of (0,1) γ = tan(π(*y*-1/2)). The Cauchy mutation operator C(γ) is obtained according to Eqs (5, 6) and is used to update the position. The formula is as follows:

(7) $$\begin{array}{cc} X_{\text{new}}\,\left(i\right)=X\,\left(i\right)+X\,\left(i\right)\times C\,\left(\gamma \right) & i\in \left\{1,\ldots ,N\right\} \end{array}.$$

#### Levy Mutation Strategy

Many organisms in nature use the Levy flight strategy when foraging for food (Faramarzi et al., 2020). Moreover, many heuristic algorithms have been improved based on this strategy and achieved good results (Zhu et al., 2013; Aydogdu et al., 2016; Li et al., 2019; Iacca et al., 2021). Levy flight has a strong disturbance capability and is a motion mode of alternate exploration through high-frequency short distance exploration and low-frequency long distance exploration. This not only expands the search range but also enhances the local search capability in a specific region. Moreover, this approach can avoid falling into the local optimal when seeking the optimal solution in a large range. Another important point is that the introduction of Levy flight can effectively avoid the excessive dependence of position changes on the position information of the previous generation, thus ensuring the diversity of the species. A simple version of the Levy distribution is mathematically defined as

(8) $$\text{levy}\,\left(\beta \right):\mu =t^{-1-\beta },0<\beta \leq 2.$$

The expressions of Levy random numbers are as follows:

(9) $$\text{levy}\,\left(\beta \right):\frac{\varphi \times \mu }{{\left|\nu \right|}^{1/\beta }},$$

(10) $$\varphi ={\left[\frac{\Gamma \,\left(1+\beta \right)\times \sin\left(\pi \times \beta /2\right)}{\Gamma \,\left(\left(\frac{1+\beta }{2}\right)\times \beta \times 2^{\frac{\beta -1}{2}}\right)}\right]}^{1/\beta },$$

where μ and ν are all standard normal distribution, β is typically 1.5, Γ is the standard gamma function.

The Levy mutation operator is applied to the GBO algorithm to update the position and the formula is as follows:

(11) $$\begin{array}{cc} X_{n\,e\,w}\,\left(i\right)=X\,\left(i\right)+X\,\left(i\right)\times L\,\left(\beta \right) & i\in \left\{1,\ldots ,N\right\} \end{array},$$

where *L*(β) is a randomly distributed number obtained from the Levy distribution. The Levy flight strategy can search in the space far enough away from the current optimal solution to ensure that individuals can jump out of the local optimal solution.

### Pseudo-Code of CLGBO


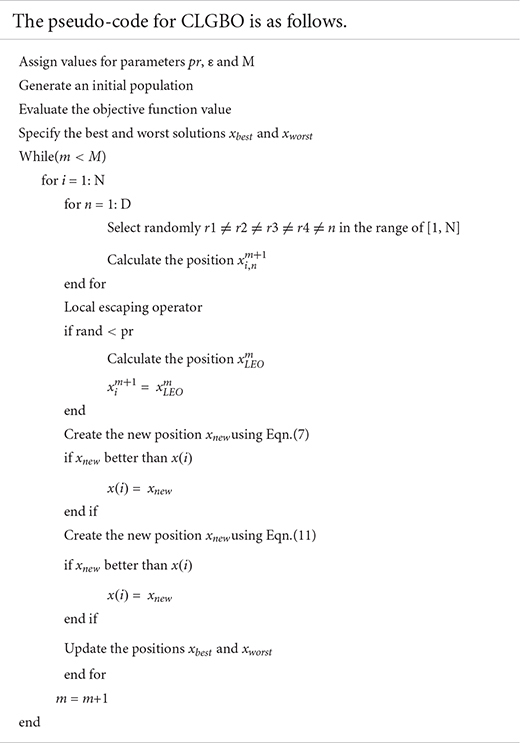


### Experimental Environment

All experimental tests were conducted in a unified environment and the detailed parameters are shown in Table 1.

**TABLE 1:** Operating environment.

Name	Value
**Hardware:**	
CPU	Core i5
Frequency	2.30 GHZ
RAM	8 GB
**Software:**	
Operating system	Windows 10
Language	MATLAB R2018b

### Benchmark Functions and Experimental Setup

The 14 benchmark functions of the famous CEC-2017 are used to comprehensively evaluate the overall performance of the CLGBO algorithm. These 14 test functions have been widely used in previous studies (Hashim et al., 2019; Faramarzi et al., 2020). The 14 test functions are divided into two categories as a benchmark to test the performance of the algorithm: one is a unimodal function (F_1_–F_6_) and the other is a multimodal function (F_7_–F_14_). The mathematical model, dimension, search space, and theoretical optimal values of all functions are listed in Tables 1, 2 in note 1 of the Supplementary Material. The CLGBO algorithm was compared with six well-known metaheuristic optimization algorithms to benchmark its performance: GBO, GWO, CS, ABC, WOA, and ISA. All of the data for the performance of these algorithms were taken from the literature (Ahmadianfar et al., 2020). In addition, all tests were conducted under same conditions. The size of the population and the maximum number of iterations were set at 50 and 500, respectively. At the same time, each test function was independently executed 30 times to reduce the randomness of the results, the best, average and standard deviation values were calculated. When solving the minimum problem, the smaller average value is, the better the algorithm performance, and a smaller standard deviation value indicates a more stable algorithm. Therefore, we use average and standard deviation values to evaluate the performance and stability of the algorithm. The specific results are shown in Tables 2,3 and bold font indicates the best results. In the following subsections, the exploitation, exploratory capability and speed of convergence of the CLGBO algorithm are analyzed. A non-parametric statistical Wilcoxon rank sum test is also conducted to further evaluate the algorithm.

**TABLE 2:** Results of the unimodal test functions.

ID	Metric	CLGBO	CGBO	LGBO	GBO	GWO	CS	ABC	WOA	ISA
F_1_	Best	**0.00E + 00**	1.15E-309	0.00E + 00	1.26E-135	4.33E-29	4.44E-05	6.25E-10	9.43E-89	2.94E + 00
	AVG	**0.00E + 00**	6.85E-309	3.61E-316	1.46E-125	3.87E-27	2.52E-02	1.77E-02	6.75E-80	9.87E + 01
	SD	**0.00E + 00**	0.00E + 00	0.00E + 00	7.96E-125	7.73E-27	1.17E-01	6.49E-02	2.45E-79	1.92E + 02
F_2_	Best	**0.00E + 00**	0.00E + 00	0.00E + 00	2.33E-206	2.79E-108	1.46E-06	1.90E-76	9.17E-141	6.76E-09
	AVG	**0.00E + 00**	0.00E + 00	0.00E + 00	3.29E-193	4.17E-97	1.81E + 01	3.76E-54	1.56E-110	1.61E-01
	SD	**0.00E + 00**	0.00E + 00	0.00E + 00	0.00E + 00	1.87E-96	8.44E + 01	2.06E-53	7.86E-110	6.00E-01
F_3_	Best	**0.00E + 00**	5.59E-311	0.00E + 00	1.50E-138	2.25E-31	5.38E-03	2.11E-08	2.88E + 01	4.16E-04
	AVG	**0.00E + 00**	1.10E-297	9.88E-324	2.40E-128	5.78E-29	9.00E-01	1.32E + 00	5.52E + 03	1.54E-02
	SD	**0.00E + 00**	0.00E + 00	0.00E + 00	1.21E-127	1.48E-28	1.70E + 00	2.68E + 00	3.85E + 03	2.88E-02
F_4_	Best	**1.87E + 01**	1.79E + 01	1.83E + 01	1.98E + 01	2.52E + 01	2.96E + 01	3.97E + 01	2.69E + 01	2.35E + 01
	AVG	2.14E + 01	**2.10E + 01**	2.16E + 01	2.16E + 01	2.68E + 01	1.39E + 02	6.93E + 01	2.75E + 01	7.56E + 01
	SD	1.41E + 00	1.32E + 00	1.86E + 00	**8.03E-01**	7.53E-01	2.37E + 02	5.50E + 01	4.12E-01	5.18E + 01
F_5_	Best	**0.00E + 00**	2.25E-309	0.00E + 00	3.92E-140	1.61E-34	6.67E-06	4.06E-16	2.63E-94	2.54E-05
	AVG	**0.00E + 00**	3.79E-298	1.98E-323	8.86E-131	5.60E-33	5.16E-04	1.56E-08	2.86E-84	1.50E-01
	SD	**0.00E + 00**	0.00E + 00	0.00E + 00	4.07E-130	5.84E-33	7.63E-04	7.60E-08	1.11E-83	7.42E-01
F_6_	Best	**0.00E + 00**	3.06E-308	0.00E + 00	1.35E-136	1.12E-31	1.22E-02	1.48E-10	2.90E-89	2.80E-02
	AVG	**0.00E + 00**	1.10E-293	1.34E-321	9.61E-129	5.14E-30	1.88E-01	6.39E + 00	1.30E-81	6.14E + 01
	SD	**0.00E + 00**	0.00E + 00	0.00E + 00	4.92E-128	8.14E-30	3.04E-01	3.50E + 01	5.59E-81	1.65E + 02

**TABLE 3:** Results of the multimodal test functions.

ID	Metric	CLGBO	CGBO	LGBO	GBO	GWO	CS	ABC	WOA	ISA
F_7_	Best	**0.00E + 00**	0.00E + 00	0.00E + 00	0.00E + 00	2.11E + 00	7.74E + 00	8.69E + 00	0.00E + 00	9.06E + 00
	AVG	**0.00E + 00**	0.00E + 00	0.00E + 00	0.00E + 00	5.91E + 00	9.86E + 00	1.05E + 01	3.00E + 00	1.09E + 01
	SD	**0.00E + 00**	0.00E + 00	0.00E + 00	0.00E + 00	2.20E + 00	8.36E-01	9.07E-01	4.43E + 00	8.96E-01
F_8_	Best	**5.70E-10**	4.68E-09	1.25E-09	4.60E-09	6.36E-01	6.28E-01	4.49E-01	6.99E-02	4.72E + 00
	AVG	**1.05E-07**	1.39E-07	7.07E-08	2.96E-07	1.01E + 00	2.41E + 00	3.84E + 00	5.12E-01	3.42E + 01
	SD	**3.68E-07**	3.07E-07	9.31E-08	8.45E-07	1.59E-01	2.27E + 00	3.98E + 00	3.58E-01	2.25E + 01
F_9_	Best	**3.82E-04**	3.82E-04	3.82E-04	3.82E-04	3.82E-04	3.82E-04	3.82E-04	3.82E-04	3.83E-04
	AVG	3.82E-04	3.82E-04	3.82E-04	3.82E-04	3.82E-04	**4.12E-04**	9.84E + 01	3.82E-04	1.87E-03
	SD	**0.00E + 00**	0.00E + 00	0.00E + 00	0.00E + 00	8.72E-13	4.54E-05	1.66E + 02	5.55E-13	5.08E-03
F_10_	Best	**8.88E-16**	8.88E-16	8.88E-16	8.88E-16	3.64E-14	4.69E-04	2.22E + 00	8.88E-16	1.33E-03
	AVG	**8.88E-16**	8.88E-16	8.88E-16	8.88E-16	4.46E-14	3.73E-03	4.90E + 00	3.73E-15	9.27E-01
	SD	**0.00E + 00**	0.00E + 00	0.00E + 00	0.00E + 00	4.19E-15	3.44E-03	1.51E + 00	2.70E-15	8.13E-01
F_11_	Best	1.42E-14	**7.11E-15**	2.84E-14	1.35E-13	2.27E + 01	8.53E-14	3.79E + 00	7.11E-15	3.46E + 01
	AVG	6.92E-14	6.70E-14	8.08E-14	1.97E-13	2.91E + 01	6.23E-02	9.29E + 00	**1.92E-14**	3.89E + 01
	SD	**2.50E-14**	2.51E-14	2.95E-14	3.62E-14	3.34E + 00	9.52E-02	3.89E + 00	6.62E-14	1.71E + 00
F_12_	Best	2.82E-01	**1.66E-01**	2.97E-01	3.46E-01	4.41E-01	4.42E-01	2.64E-01	2.60E-01	3.31E-01
	AVG	4.93E-01	4.88E-01	4.95E-01	5.31E-01	6.39E-01	5.93E-01	5.19E-01	5.24E-01	**4.63E-01**
	SD	1.22E-01	1.23E-01	1.14E-01	1.72E-01	9.60E-02	8.40E-02	1.84E-01	1.88E-01	**9.80E-02**
F_13_	Best	4.91E-01	4.97E-01	5.00E-01	4.06E-01	3.43E-01	3.18E-01	2.25E-01	**1.21E-01**	2.54E-01
	AVG	4.96E-01	5.00E-01	4.90E-01	4.24E-01	4.65E-01	4.54E-01	5.78E-01	**3.84E-01**	6.42E-01
	SD	1.93E + 00	**6.29E-04**	1.89E + 00	1.17E-02	7.42E-02	1.47E-01	2.71E-01	9.68E-02	2.96E-01
F_14_	Best	2.03E-243	1.18E-158	9.98E-172	2.95E-73	1.64E-19	2.60E-05	4.20E-18	**0.00E + 00**	1.27E-04
	AVG	3.92E-236	1.21E-153	1.40E-164	6.45E-69	4.27E-04	2.64E-02	1.74E-03	**0.00E + 00**	6.15E-01
	SD	0.00E + 00	4.83E-153	0.00E + 00	1.99E-68	5.72E-04	2.69E-02	9.45E-03	**0.00E + 00**	1.22E + 00

### Experimental Results

#### Evaluation of the Exploitation Ability

Unimodal functions (F_1_–F_6_) are usually used to evaluate the exploitation ability of the optimization algorithm. These test functions have only one global optimal solution and no local optimal solution. They can therefore be used to evaluate the exploitation capability of the CLGBO algorithm. The results of the CGBO (LGBO) algorithm obtained by adding only the Cauchy (Levy) mutation operator are shown in Table 2. The CGBO and LGBO algorithms have improved in all three test metrics (best value, average, and standard deviation), but are inferior to the CLGBO algorithm, which proves that the combination of the two mutation operators is more effective than using only one of them. For example, the mean value of function F_1_ is reduced by more than 100 orders of magnitude by only using one of the mutation strategies, but neither of them converges to the global minimum value 0. When the two mutation strategies are combined, the average value converges to the global optimal value 0. The best value (Best), average (AVG), and standard deviation (SD) of the test functions F_1_–F_3_ and F_5_–F_6_ for the CLGBO algorithm have reached the global optimal value. The F_4_ function does not reach the global optimal value. However, its optimal value and average value are improved compared with the original GBO algorithm. The average value of 5 of the 6 unimodal test functions is 0, which proves that the algorithm converges to the global optimum in different mathematical models, and their variances are also 0, which proves that the data has strong stability. Compared with the other six optimization algorithms, the CLGBO algorithm has obvious advantages in the exploitation stage.

#### Evaluation of the Exploration Ability

The exploration ability of the CLGBO algorithm is evaluated by multimodal functions (F_7_–F_14_). These functions have a global optimal solution and a large number of local optimal solutions. The number of local optimal solutions increases exponentially as the dimensions of the problems increase. Therefore, the multimodal functions can reflect well the exploration ability of the algorithm. The results of the CGBO (LGBO) algorithm obtained by simply adding the Cauchy (Levy) mutation operator are listed in Table 3. The three test indexes (the best value, average, and standard deviation) of the CGBO and LGBO algorithms are improved but they are not as good as the CLGBO algorithm. This proves once again that the combination of mutation operators is more effective than using only one of these operators alone. The function F_7_ also reaches the global optimal value in CLGBO. The average of the function F_8_ is closer to the global optimal value than the other six algorithms. Its standard deviation is smaller than the other algorithms, which indicates that the results of the CLGBO algorithm are more stable. The functions F_9_, F_10_, and F_13_ in CLGBO are almost identical to the results in GBO. F_11_ and F_12_ are not the best results for these seven algorithms. However, they are significantly better than the previous GBO results. The results show that CLGBO has strong exploration ability.

#### Evaluation of Convergence Efficiency

**FIGURE 3:**
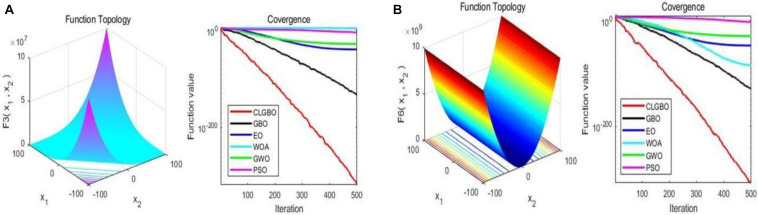
3D representation and convergence curve of two unimodal functions. **(A)** the result of the function F3. **(B)** the result of the function F6.

**FIGURE 4:**
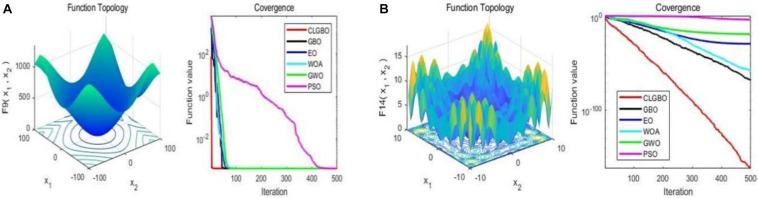
3D representation and convergence curve of two multimodal functions. **(A)** the result of the function F9. **(B)** the result of the function F14.

The convergence curve is an important indicator for the performance of the algorithm, through which we can see the convergence speed and the ability of the algorithm to jump out of the local optimum. For further illustration, the convergence curves of the CLGBO and other 5 algorithms are plotted in Figures 3, 4. Figures 3, 4 contain three-dimensional representations and convergence curve of unimodal functions (F_3_, F_6_) and multimodal functions (F_9_, F_14_). The remaining three-dimensional representation of unimodal and multimodal functions and convergence curves can be found in note 2 of the Supplementary Material. All optimization algorithms hope to achieve global optimization quickly and accurately. Convergence curves are often used to evaluate the convergence efficiency of an algorithm. The changes of convergence curves of the GBO, EO, WOA, GWO, and PSO algorithms are also depicted in Figures 3, 4. The convergence speed of the CLGBO algorithm is faster than the speed of the other five algorithms, which is clear from the convergence curves in Figures 3, 4. This is true for both the unimodal and multimodal functions and indicates that the CLGBO algorithm can achieve an appropriate balance between exploration and exploitation. More importantly, the convergence curves can reach the global optimal value accurately in the optimization process of the CLGBO algorithm.

#### Wilcoxon Rank Sum Test

The Wilcoxon rank sum test (Kim and Kim, 1996) was used to evaluate the significant difference between the two positions of the CLGBO algorithm. The test randomly selects two sets of samples and the *P*-values obtained can be used as an indicator for evaluating the algorithm. Specifically, the corresponding algorithm is considered to have a statistically significant advantage when the *P*-values are greater than 0.05.

We ran each algorithm 30 times and calculated its average value to avoid the randomness of the results. The *P*-values obtained by the 14 test functions from this statistical test are shown in Tables 4, 5. The *P*-values of the CLGBO algorithm are greater than 0.05, which indicates that this algorithm provides very competitive results.

**TABLE 4:** *P*-values of Wilcoxon rank sum test.

	F_1_	F_2_	F_3_	F_4_	F_5_	F_6_
GBO	0.47379	0.48579	0.52822	0.56719	0.51674	0.48343
CLGBO	0.51016	0.50425	0.57822	0.60641	0.59674	0.50238

**TABLE 5:** *P*-values of Wilcoxon rank sum test.

	F_7_	F_8_	F_9_	F_10_	F_11_	F_12_	F_13_	F_14_
GBO	0.4922	0.44528	0.46159	0.6046	0.51303	0.53191	0.57046	0.57868
CLGBO	0.48824	0.47581	0.48725	0.48092	N/A	0.56072	0.70443	0.55438

## Designing of Lower Bounds of Coding Sets

The construction of DNA storage coding sets that satisfy constraints can be used as primer (address) libraries. These constructed coding sets are essential to enable random storage. It has been shown in the literature (Organick et al., 2018) that each file can be recovered individually without error using a random-access method. Restricted by the existing DNA synthesis technology, the encoded base sequence will be divided into short sequences of the same length, and the length of a single sequence is generally no more than 200 bp. Each sequence that needs to be synthesized includes primers, data, address bits, and error-correcting codes, etc., among which address bits are used for quick positioning, stitching and searching of each sequence. Primers are specially designed and added to both ends of the sequence prior to synthesis to extract the desired DNA sequence. We can obtain the content of this file by adding primers to the DNA pool using PCR technology for amplification, and subsequently sequencing and decoding. With the development of random DNA storage, primers, and address bits play important roles. Therefore, it is very essential to construct more and highly robust DNA coding sets as primer (address) libraries.

### The Comparison of Lower Bounds

In this study, we apply the CLGBO algorithm to practical problems to improve the lower bounds of coding sets. A^*GC, NL*^ (*n*, *d*, and *w*) represents the sets of DNA sequences that satisfy the GC content constraint, the No-runlength constraint and the Hamming distance constraint, where *n* represents the length of the sequence, *d* represents the size of the Hamming distance and *w* represents the GC content, which is usually *n*/2. Meanwhile, we compared results for CLGBO algorithm with the best results recently obtained using the altruistic algorithm proposed by Limbachiya et al. (2018) and the NOL-HHO algorithm used by Yin et al. (2020). Altruistic algorithm is an intelligent algorithm which uses greedy algorithm to iteratively delete potential code words. It removes the “worst” candidate code word in each iteration. NOL-HHO algorithm is an algorithm to improve the Harris Hawks optimization algorithm by using a new nonlinear control parameter strategy and a random opposition-based learning strategy. Tables 6, 7 show the lower bounds of coding sets of 4≤*n*≤10, 3≤*d*≤*n* obtained using the altruistic algorithm and NOL-HHO algorithm, respectively. Table 8 shows the lower bounds of the coding sets using the CLGBO algorithm. The black bold font indicates the optimal result and the numbers in parentheses represent the best lower bounds of the coding sets acquired by the altruistic algorithm and the NOL-HHO algorithm. The superscripts are identified in Table 9.

**TABLE 6:** Lower bounds of the altruistic algorithm for A^*GC, NL*^ (*n*, *d*, and *w*; Limbachiya et al., 2018).

*n*\*d*	3	4	5	6	7	8	9
4	11						
5	17	7					
6	44	16	6				
7	110	36	11	4			
8	289	86	29	9	4		
9	662	199	59	15	8	4	
10	1810	525	141	43	7	5	4

**TABLE 7:** Lower bounds of the NOL-HHO algorithm for A^*GC, NL*^ (*n*, *d*, and *w*; Yin et al., 2020).

*n*\*d*	3	4	5	6	7	8	9
4	12						
5	20	8					
6	55	23	8				
7	121	42	14	7			
8	339	108	35	13	5		
9	705	216	69	22	11	4	
10	1796	546	148	51	20	9	4

**TABLE 8:** Lower bounds of the CLGBO algorithm for A^*GC, NL*^ (*n*, *d*, and *w*).

*n*\*d*	3	4	5	6	7	8	9
4	**12**^*c*^ (12^*n*^)						
5	**20**^*c*^ (20^*n*^)	**8^*c*^** (8^*n*^)					
6	**58**^*c*^ (55^*n*^)	**24^*c*^** (23^*n*^)	**8^*c*^** (8^*n*^)				
7	**131**^*c*^ (121^*n*^)	**45**^*c*^ (42^*n*^)	**17^*c*^** (14^*n*^)	**7^*c*^** (7^*n*^)			
8	**349**^*c*^ (339^*n*^)	**113**^*c*^ (108^*n*^)	**38^*c*^** (35^*n*^)	**15**^*c*^ (13^*n*^)	**5**^*c*^ (5^*n*^)		
9	**743**^*c*^ (705^*n*^)	**234**^*c*^ (216^*n*^)	**71^*c*^** (69^*n*^)	**27**^*c*^ (22^*n*^)	**11**^*c*^ (11^*n*^)	**5**^*c*^ (4^*a, n*^)	
10	**2030**^*c*^ (1810^*a*^)	**580**^*c*^ (546^*n*^)	**168^*c*^** (148^*n*^)	**56**^*c*^ (51^*n*^)	**23^*c*^** (20^*n*^)	**9**^*c*^ (9^*n*^)	**5**^*c*^ (4^*a, n*^)

**TABLE 9:** Meaning of superscript.

Superscript	Meaning
a	Altruistic algorithm (Limbachiya et al., 2018)
n	NOL-HHO algorithm (Yin et al., 2020)
c	CLGBO algorithm

The lower bounds of the coding sets acquired using the CLGBO algorithm are higher than the other two algorithms (Table 8). The multiple coding sets reported in the table are in the same state as previous work, for example, *n* = 4,5, *d* = 3; *n* = 7, *d* = 6. This is mainly the case since we have reached the limit of the number of sequences that satisfy the constraint, which is the theoretically optimal value. However, the lower bound acquired using the CLGBO algorithm improves significantly further for the same value of *d* as *n* increases. For example, when *d* = 3, *n* = 6, 7, 8, 9, and 10, the lower bounds of the coding sets obtained by CLGBO algorithm are 8.6–29.5% higher than the altruistic algorithm. When *d* = 4, *n* = 6, 7, 8, 9, and 10, the lower bounds obtained using the CLGBO algorithm are 4.3–7.4% higher than the NOL-HHO algorithm. In conclusion, the CLGBO algorithm can greatly increase the number of DNA coding sets and create conditions for storing large files. In addition, the increase of the lower bounds of the coding sets directly leads to improvements of the coding rate. The coding rate is defined as R = log_4_*^*M*^*/*n* (Cao et al., 2020), where *n* is the length of coded DNA and *M* is the number of the DNA coding set. For example, the values used in previous work (Limbachiya et al., 2018) are *n* = 9 and *d* = 4, *R* = log_4_^199^/9 ≈ 0.42. When *n* = 8, *d* = 4, the encoding rate also reaches 0.42 using our algorithm. Short sequences can therefore achieve the same storage performance as long sequences at the same coding rate.

### Introduction of the Non-adjacent Subsequence Constraint

The sequence that contains consecutive repetitive subsequences is more prone to errors in the sequencing process, we propose an original constraint (non-adjacent subsequence constraint) for this, so that the constructed DNA coding sets can be more robust. The higher the robustness of the DNA coding sets, the lower the probability of errors in the DNA storage process. Therefore the non-adjacent subsequence constraint is added to the three basic constraints to build more stable and robust coding sets. The results are shown in Table 10. A^*GC, NL, NS*^ (*n*, *d*, and *w*) denotes DNA coding sets that satisfy the GC content, No-runlength, Hamming distance and non-adjacent subsequence constraints. In addition, in note 3 of the Supplementary Material, 66 sequences constructed using CLGBO when *n* = 9 and *d* = 5 are presented as experimental samples for detection.

**TABLE 10:** Lower bounds for A^*GC, NL, NS*^ (*n*, *d*, and *w*).

*n*\*d*	3	4	5	6	7	8	9	10
6	51	22	8					
7	113	42	15	6				
8	319	105	35	15	5			
9	635	206	66	25	10	5		
10	1634	518	157	56	21	10	5	
11	2974	922	282	104	38	16	8	4
12	6184	1736	577	182	68	30	14	7
13	13590	3923	1050	386	130	50	24	10

The validity of the non-adjacent subsequence constraint was tested by calculating the variance of the melting temperature of the DNA coding sets. In a DNA set, the melting temperature (Tm) of the DNA sequence is the point when 50% of the DNA double-stranded molecules become single-stranded structures due to the process of heating and deformation (Sager and Stefanovic, 2005). The Tm will affect the rate of reactions between DNA molecules in PCR amplification. Non-specific hybridization is related to the structure of oligonucleotides and their thermodynamic properties. Significantly, each oligonucleotide in the library must have a similar Tm to reduce the possibility of non-specific hybridization of the oligonucleotide library (Chee and Ling, 2008). Therefore, each sequence must have a similar Tm when designing DNA coding sets. The variance is used to value the quality of sequences: the smaller the variance, the more stable the Tm of the whole coding set.

**FIGURE 5:**
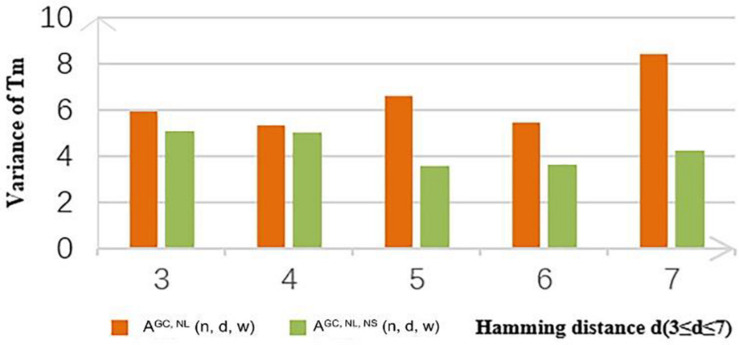
Tm difference of A^*GC, NL*^ (*n*, *d*, and *w*) and A^*GC, NL, NS*^ (*n*, *d*, and *w*) with *n* = 8.

In this study, the concentration of the DNA molecule was set at 10 nM and the concentration of salt was set at 1 M. Coding sets with and without the new constraint obtained by the CLGBO algorithm were analyzed for their Tm values. As can be seen from the values in Table 11, 93% of the values show that the variance with the constraint is smaller than that without the constraint. In addition, the Tm variance of coding sets obtained by adding the new constraint were reduced by 10–66% compared with the values obtained without adding this constraint (Table 11). The Tm values of the sequences in a coding set are closer if the Tm variance was smaller. To highlight our results, a comparison between the variances of Tm obtained for A^*GC, NL*^ (*n*, *d*, and *w*) and A^*GC, NL, NS*^ (*n*, *d*, and *w*) when *n* = 8 are shown in Figure 5. The variance of Tm for the coding sets with this constraint is smaller than without this constraint. And when *n* = 8, *d* = 7, the variance of the coding set with or without this constraint differs by 4.1535. When the variance of the coding set TM value is small, the possibility of non-specific hybridization is reduced and the PCR reaction is more stable. At the same time, the results confirm the applicability and necessity of the non-adjacent subsequence constraint.

**TABLE 11:** Comparison of the variance of Tm.

n\d		3	4	5	6	7
8	A^*GC, NL*^	5.9351	5.2979	6.6136	5.4233	8.3799
	A^*GC, NL, NS*^	**5.0461**	**4.9945**	**3.5621**	**3.5888**	**4.2264**
9	A^*GC, NL*^	4.7033	4.8000	4.7655	3.6546	4.8876
	A^*GC, NL, NS*^	**4.5800**	**4.4041**	**3.9916**	**2.8110**	**1.4578**
10	A^*GC, NL*^	4.4705	4.5131	4.8233	5.0288	**3.3062**
	A^*GC, NL, NS*^	**4.0754**	**4.0554**	**4.2452**	**4.2037**	3.9066

*The bold values indicates the smaller the variance of TM.*

## Conclusion

In this study, the CLGBO algorithm and non-adjacent subsequence constraint were combined to construct more stable primer and address libraries for DNA storage. First, the GBO algorithm was improved by employing the Cauchy mutation operator and Levy strategy. Cauchy mutation operator not only expands the diversity of the population, but also can effectively reduce the search blind spots, improve the exploration ability and convergence speed of the algorithm. Levy flight strategy can effectively avoid the over-dependence of position update on the previous position, and search for the optimal solution in a large range, so as to avoid falling into local optimum and premature convergence. The combination of the two strategies not only controlled the global ability well but also enhanced the local exploration ability, and makes the algorithm achieve a good balance in the exploitation and exploration stages. Next, the classical CEC-2017 test function and the Wilcoxon rank sum test were adopted to evaluate comprehensively the CLGBO algorithm in the exploitation phase, exploration phase and statistically. The test results and convergence curves showed that the CLGBO algorithm has stronger competitiveness, convergence ability and optimization ability compared with other algorithms. Second, CLGBO algorithm was applied to construct DNA storage coding sets. The lower bounds of DNA coding sets constructed by the CLGBO algorithm under the same constraint were significantly increased by 4.3–13.5% compared with previous work, and there was also an improvement of the coding rate. When storing large files, it is possible to use shorter DNA primers and address sequences due to the improved lower bounds of the coding sets. Shorter DNA sequences mean lower error rates for DNA synthesis and sequencing. Finally, sequences containing consecutive repetitive subsequences are prone to cause errors during DNA storage. We therefore introduced the non-adjacent subsequence constraint to avoid mistakes and improve the stability of the coding sets. A comparison of the variance of Tm with and without this constraint showed that the variance of Tm with this constraint was reduced by 10–66%. The smaller Tm variance indicated that the Tm values of sequences in a DNA coding set were relatively similar. This can reduce the incidence of non-specific hybridization in the storage process and ensure that the DNA sequence is untied at similar temperatures during the PCR process to successfully amplify the DNA sequence.

In future work, we will further improve the lower bounds of the primer and address libraries while ensuring high robustness of the DNA coding sets. However, the quality of coding sets is inversely proportional to the quantity. It therefore remains a challenge to find the right balance between quality and quantity in future work. We will also continue to explore DNA storage and hope to come up with an original way of encoding for the payload and non-payload that will reduce redundancy and ensure accurate information recovery. In addition, the constructed coding sets can also be applied to other fields, including DNA image encryption (Zhou et al., 2020), DNA-binding proteins (Zhao et al., 2012), DNA computing (Li et al., 2020), and information security (Zhang et al., 2020).

## Data Availability Statement

The raw data supporting the conclusions of this article will be made available by the authors, without undue reservation.

## Author Contributions

YZ: conceptualization, resources, and writing—original draft preparation. JW: investigation. BW: writing—review, editing, and funding acquisition. All authors have read and agreed to the published version of the manuscript.

## Conflict of Interest

The authors declare that the research was conducted in the absence of any commercial or financial relationships that could be construed as a potential conflict of interest.
