# Supplementary material for: CLGBO: An Algorithm for Constructing Highly Robust Coding Sets for DNA Storage
Source: Front Genet. 2021 May 4;12:644945. doi: 10.3389/fgene.2021.644945 (PMC8129200; doi:10.3389/fgene.2021.644945)
Supplement: Supplementary file 1 [file Data_Sheet_1.docx]

Supplementary Material

This supplement document is mainly used to supplement the paper (CLGBO: An algorithm for constructing highly robust coding sets for DNA storage). This document most importantly includes mathematical models of 14 test functions, three-dimensional representations of 10 test functions, comparison graphs of convergence curves between CLGBO algorithm and other five algorithms, and a coding set (*n*=9, *d*=5) constructed by CLGBO algorithm. The 14 test functions are mainly divided into 6 unimodal functions and 8 multimodal functions. These unimodal functions (f_1_-f_6_) have only one global optimal solution and no local optimal solution. They can therefore be used to evaluate the exploitation capability of the CLGBO algorithm. These unimodal functions (f_7_-f_14_) have a global optimal solution and a large number of local optimal solutions. Therefore, the multimodal functions can reflect well the exploration ability of the algorithm. According to the convergence curves of the 6 algorithms in Figures1 and 2, it can be seen that the convergence speed of the CLGBO algorithm is faster than the speed of the other five algorithms and can converge to the global optimal solution in multiple functions. The coding set is constructed by CLGBO algorithm combined with 4 constraints (Hamming distance, GC content, No-runlength constraint, non-adjacent subsequence constraint), and it contains 66 sequences as test samples.

# Supplementary Note 1. Benchmark Functions.

Table 1. Description of Unimodal benchmark functions(F_1_-F_6_)

| Function | D | Range | $f_{\min}$ |
| --- | --- | --- | --- |
| $f_{1}(x)=x_{1}^{2}+10^{6}\sum_{i=2}^{D} x_{i}^{2}$ | 30 | [-100,100] | 0 |
| $f_{2}(x)=\sum_{i=1}^{D} \vert x_{i}\vert^{i+1}$ | 30 | [-100,100] | 0 |
| $f_{3}(x)=\sum_{i=1}^{D} x_{i}^{2}+(\sum_{i=1}^{D} 0.5x_{i})^{2}+(\sum_{i=1}^{D} 0.5x_{i})^{4}$ | 30 | [-100,100] | 0 |
| $f_{4}(x)=\sum_{i=1}^{D-1} [100(x_{i+1}-x_{i}^{2})^{2}+(x_{i}-1)^{2}]$ | 30 | [-100,100] | 0 |
| $f_{5}(x)=10^{6}x_{1}^{2}+\sum_{i=2}^{D} x_{i}^{2}$ | 30 | [-100,100] | 0 |
| $f_{6}(x)=\sum_{i=1}^{D} (10^{6})^{\frac{i-1}{D-1}}x_{i}^{2}$ | 30 | [-100,100] | 0 |

Table 2. Description of Multimodal benchmark functions (F_7_-F_14_).

| Function | D | Range | $f_{\min}$ |
| --- | --- | --- | --- |
| $\begin{matrix} f_{7}(x)=g(x_{1},x_{2})+g(x_{2},x_{3})+...+g(x_{D-1},x_{D})+g(x_{D},x_{1}) \\ g(x,y)=0.5+\frac{(\sin^{2}(\sqrt{x^{2}+y^{2}})-0.5)}{(1+0.001(x^{2}+y^{2}))^{2}} \end{matrix}$ | 30 | [-100,100] | 0 |
| $\begin{matrix} f8(x)=\sin^{2}(\pi w_{1})+\sum_{i=1}^{D-1} (w_{i}-1)^{2}[1+10\sin^{2}(\pi w_{i}+1)]+ \\ (w_{D}-1)^{2}[1+\sin^{2}(2\pi w_{D})] \\ \begin{matrix} where & w_{i}=1+\frac{x_{i}-1}{4} \end{matrix} \end{matrix}$ | 30 | [-100,100] | 0 |
| $\begin{matrix} \begin{matrix} f_{9}(x)=418.9829\times D-\sum_{i=1}^{D} g(z_{i}), & z_{i}=x_{i}+4.209687462275036e+002 \end{matrix} \\ g(z_{i})=\left\{\begin{matrix} z_{i}\sin(\vert z_{i}\vert^{\frac{1}{2}}) & if\vert z_{i}\vert\leq500 \\ (500-\mathrm{mod}(z_{i},500))\sin(\sqrt{\vert500-\mathrm{mod}(z_{i},500)\vert})-\frac{(z_{i}-500)^{2}}{10000D} & ifz_{i}>500 \\ (\mathrm{mod}(\vert zi\vert,500)-500)\sin(\sqrt{\left\vert\mathrm{mod}(\vert zi\vert,500)-500\right\vert})-\frac{(z_{i}+500)^{2}}{10000D} & ifz_{i}<-500 \end{matrix}\right. \end{matrix}$ | 30 | [-100,100] | 0 |
| $f_{10}(x)=20-20\times\exp(-0.2\sqrt{\frac{1}{D}(\sum_{i=1}^{D} x_{i}^{2})})-\exp(\frac{1}{D}\sqrt{\sum_{i=1}^{D} \cos(2\pi x_{i})})+e$ | 30 | [-32,32] | 0 |
| $\begin{matrix} f_{11}(x)=\sum_{i=1}^{D} \left(\sum_{k=0}^{k\max} [a^{k}\cos(2\pi b^{k}(x_{i}+0.5))]\right)-D\sum_{k=0}^{k\max} [a^{k}\cos(2\pi b^{k}.0.5)] \\ a=0.5,b=3,k\max=20 \end{matrix}$ | 30 | [-100,100] | 0 |
| $f_{12}(x)=\vert\sum_{i=1}^{D} x_{i}^{2}-D\vert^{\frac{1}{4}}+(0.5\sum_{i=1}^{D} x_{i}^{2}+\sum_{i=1}^{D} x_{i})/D+0.5$ | 30 | [-100,100] | 0 |
| $f_{13}(x)=\vert(\sum_{i=1}^{D} x_{i}^{2})^{2}-(\sum_{i=1}^{D} x_{i}^{2})^{2}\vert^{\frac{1}{2}}+(0.5\sum_{i=1}^{D} x_{i}^{2}+\sum_{i=1}^{D} x_{i})/D+0.5$ | 30 | [-100,100] | 0 |
| $f_{14}(x)=\sum_{i=2}^{D} \vert x_{i}\sin(x_{i})+0.1x_{i}\vert$ | 30 | [-100,100] | 0 |

# Supplementary Note 2. Convergence curve comparison charts.


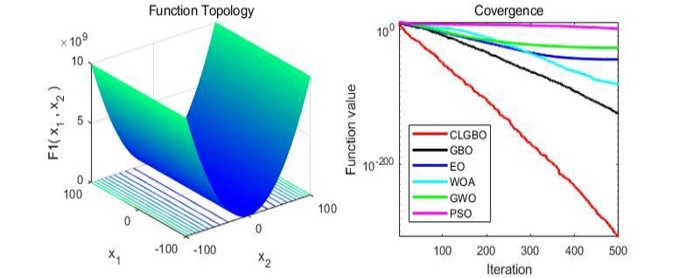

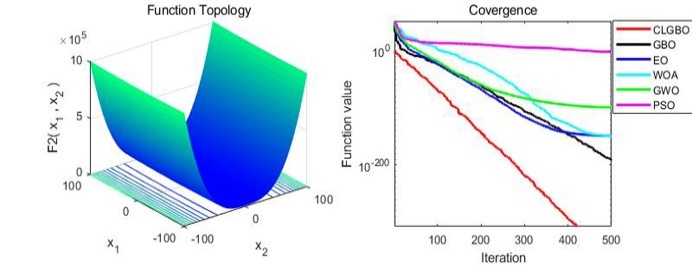


1. **(B)**


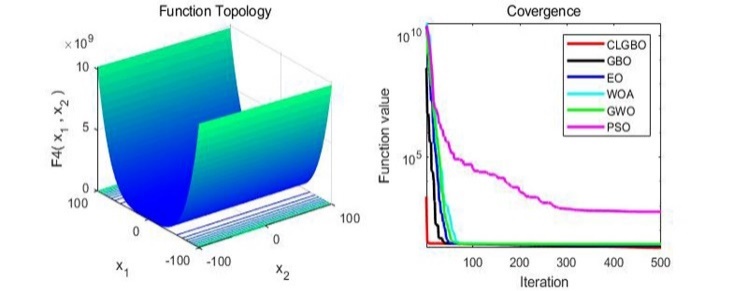
**
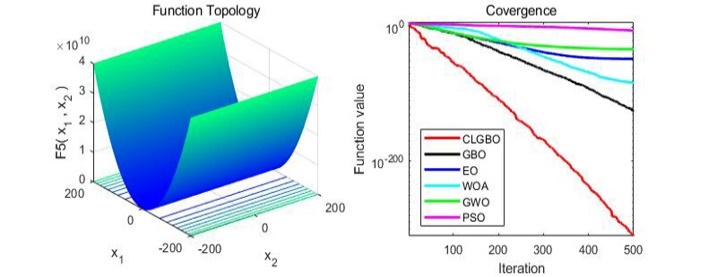
**

**(C) (D)**

Figure 1. 3D representation and Convergence curve of four unimodal functions


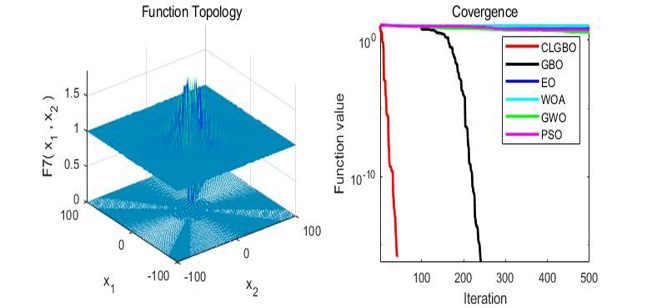

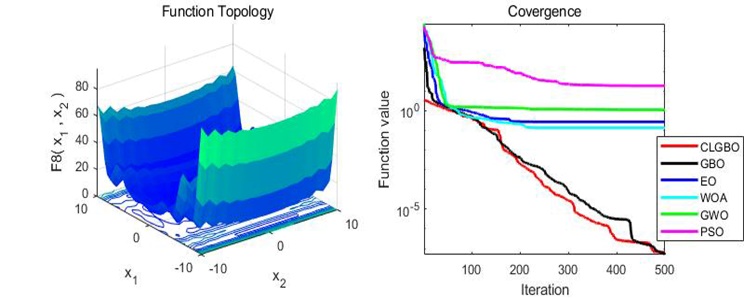


**(A) (B)**

**
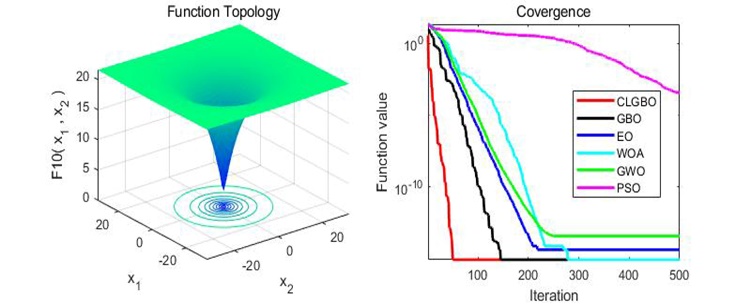

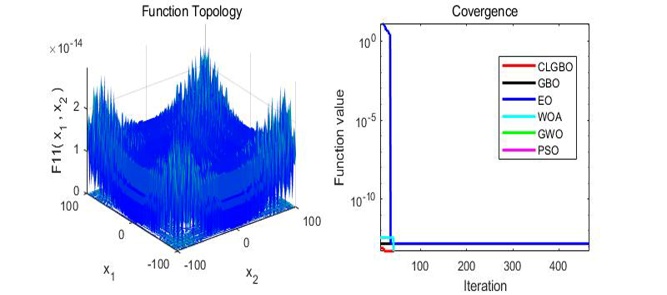
**

**(C) (D)**

**
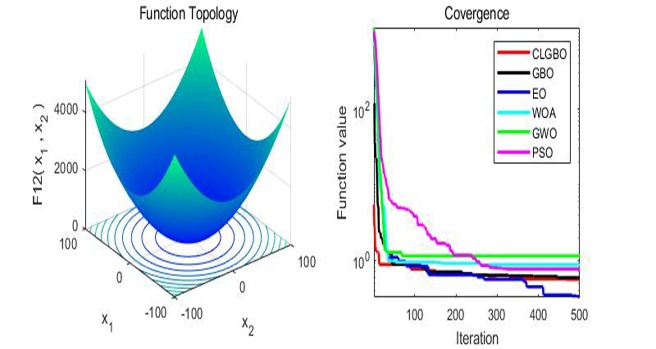

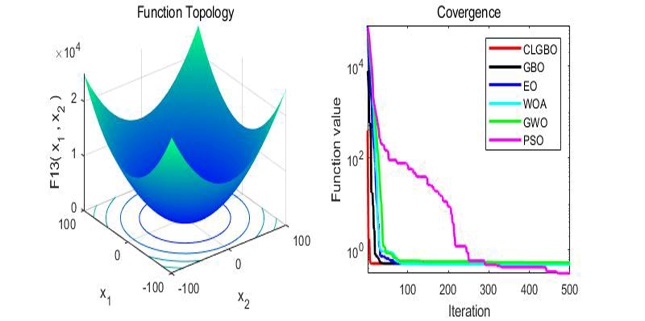
**

**(E) (F)**

Figure 2. 3D representation and Convergence curve of six multimodal functions

# Supplementary Note 3. Coding set (*n*=9, *d*=5)

Table 3. The coding set of n=9, d=5.

| ATCAGTGAG TCATGCTAC | GATGACAGT GATGACAGT CTACGATCT | TCACTACAG | TATCGTGCA | CGTCTAGAT | GTATCGTGA |
| --- | --- | --- | --- | --- | --- |
| TCATGCTAC CACTCAGTA | CTACGATCT ATGTCTGCT | CAGCATACT | TACTCGTCT | ATAGACGCA | CTCATACTG |
| GTCATGAGT | ATGATCGTC ATGCTACGT | TCTACATGC | GCTACTGAT | CATGTCTAC | AGTACAGCA |
| CACTCAGTA ATAGCACTC TAGTGACGA | ATGTCTGCT CAGATCAGA | ACATGAGTG | TCGTATGTC | GAGTACTCA | GCTATCATG |
| CTGTCATAC | CATACGATC | ATGTAGCTG | CATAGTCGT | AGATGCAGA | ATACTGACG |
| TCATAGCGT | ATGCTACGT | ACTATGCAC | CTACATGAC | TGCATCTAG | TCGATGTCA |
| ACTCACTAG TCATGCTAC | CTCACTACA GATGACAGT CTACGATCT | CTAGTGCAT | GTCTACATC | AGACGTCAT | GATAGATCG |
| ATAGCACTC CACTCAGTA | CAGATCAGA ATGTCTGCT | TGCAGTCTA | GTCGTATCA | AGCTACGAT | ACTCGTATC |
| TAGTGACGA TCATAGCGT | TGACTCGTA ATGCTACGT | AGCAGATGT | TGATCTGAG | TCAGCTAGA | AGCGTGATA |
| TACGATCAG ATAGCACTC TAGTGACGA | TGTAGCACT CAGATCAGA | GAGTCTATG | CATCAGTGA | GAGCTGTAT | ACAGTCTGT |
| GCTGATCTA | GTCTGACAT | ATCGAGTAC | GATCTACTC | AGTGATACG | GCATGTACT |

For example, when *n*=9 and *d*=5, the coding set is as follows:
